# Associations between carbon monoxide exposure, physical activity, and outdoor play preferences among UK children: evidence from the millennium cohort study

**DOI:** 10.1186/s12889-026-28149-8

**Published:** 2026-06-27

**Authors:** Mona Kuroiwa, Hei Wan Mak, Jitka Pikhartova, Celine Tabche, Nicola Shelton

**Affiliations:** 1 School of Public Health, Imperial College London, London, UK; 2 Department of Behavioural Science & Health, University College London, London, UK; 3 Department of Epidemiology and Public Health, University College London, London, UK; 4 Population Health Sciences, University College London, London, UK

**Keywords:** Physical activity, Carbon monoxide, Children, Accelerometry, Outdoor play, Millennium Cohort study, Air pollution, Built environment, Public health

## Abstract

**Background:**

Physical inactivity and air pollution are significant global health concerns, yet limited evidence examines how residential air pollution is associated with physical activity behaviours among children. Existing research has mainly focused on adults, older adults, or self-reported activity outcomes. This study examined the association between residential carbon monoxide (CO) exposure, objectively measured physical activity, and children’s reported preference for outdoor sports and games using data from the Millennium Cohort Study.

**Methods:**

This study used a cross-sectional secondary analysis of Sweep 4 Millennium Cohort Study data. Physical activity was measured using accelerometer-derived total steps over one week. Residential CO exposure was derived from linked MEDIx air pollution deciles and categorised into relatively lower and higher exposure groups. Linear regression was used to examine the association between CO exposure and total steps. Logistic regression was used to examine the association between CO exposure and preference for outdoor sports and games. Models were adjusted for sex, ethnicity, and socio-economic position, with survey and non-response weights applied.

**Results:**

The analytic sample included 6,281 children. In the adjusted linear regression model, children living in areas with higher CO exposure recorded, on average, 356.26 more steps than those living in areas with lower CO exposure. In the adjusted logistic regression model, higher CO exposure was associated with slightly higher odds of reporting a preference for outdoor sports and games. However, the adjusted step-count model explained only a small proportion of variation in total steps.

**Conclusion:**

Higher residential CO exposure was modestly associated with higher total steps and greater reported preference for outdoor sports and games. These findings should be interpreted cautiously because the study was cross-sectional, explanatory power was low, and residual confounding by neighbourhood, household, school travel, and built-environment factors is likely. Future studies should examine multiple pollutants alongside more detailed contextual and environmental measures.

**Supplementary Information:**

The online version contains supplementary material available at https://doi.org/10.1186/s12889-026-28149-8.

## Background

Physical inactivity and air pollution are major global public health challenges, each contributing substantially to the burden of disease [1]. In the United Kingdom (UK), just over half of the 6-year-old children (53%) meet the recommended physical activity guidelines [2]. At the same time, air pollution remains a widespread concern, with 9 out of 10 people worldwide living in areas where air pollution levels exceed the World Health Organization (WHO) air quality guidelines [3]. Given the importance of both physical activity and air pollution for health, understanding how environmental exposures are associated with children’s physical activity is of clear relevance for research and policy. As healthy behaviours and lifestyle patterns are often established early in life, identifying environmental and social factors linked to children’s physical activity is particularly important [4].

Research has suggested that air pollution, in the context of physical activity, may reduce or alter the health benefits of being active outdoors [5]. Exposure to air pollution has been associated with decreased lung function, elevated blood pressure, and other cardiovascular and respiratory outcomes, which may impair exercise tolerance and performance [6, 7]. However, the relationship between air pollution and physical activity is complex and not solely determined by physiological mechanisms. Areas with higher pollutant exposure, such as urban or traffic-dense environments, may also differ in walkability, access to destinations, neighbourhood design, school travel patterns, and opportunities for outdoor play. These contextual factors may independently influence physical activity behaviours, complicating the interpretation of observed associations.

Despite this complexity, the existing evidence base remains unevenly distributed across population groups. Most studies examining the association between air pollution and physical activityhave focused on adults and older populations, with comparatively limited attention given to children. A mapping review by Tainio et al. [8], found that the majority of included studies focused on adults, while a few focused on older adults. Evidence among children is therefore limited. One recent study conducted in China, found that highly daily examined the relationship between ambient air pollution, physical activity, and sedentary behaviour among children and found that higher daily Air Quality Index levels were associated with lower moderate-to-vigorous physical activity, fewer walking steps, and greater sedentary behaviour among children [9]. Further research is needed to better understand these associations in younger populations, particularly using objective measures of physical activity.

In addition, demographic and socio-economic characteristics are likely to play an important in this context, as they may influence both exposure to environmental risk and opportunities for physical activity. Previous research has highlighted differences in physical activity by sex and ethnicity [10] alongside broader social inequalities in environmental exposure [11, 12]. Children’s activity patterns may vary according to household income, neighbourhood conditions, access to safe outdoor spaces, school travel routines, and family resources, all of which may interact with environmental exposures such as air pollution.

Although the linked MEDIx dataset includes several pollutants, including particulate matter (PM10), nitrogen dioxide (NO₂), ozone (O₃), sulphur dioxide (SO₂), and carbon monoxide (CO), this study focuses on CO. This is because the available CO exposure data (2001–2006) most closely align with the Millennium Cohort Study (MCS) Sweep 4 physical activity data collection period [13]. CO may also serve as an indicator of combustion-related and traffic-related exposures, which overlap with environmental features relevant to walking, school travel, and outdoor play. As such, this study should be interpreted as an exploratory pollutant-specific analysis rather than a comprehensive assessment of all ambient air pollutants.

Previous research has also differed in how exposure and outcome measures are assessed. For example, Wells et al., [12] examined self-reported changes in activity in response to perceived or reported poor air quality using data from the National Health and Nutrition Examination Survey. In contrast, the present study links residential annual mean CO exposure to objectively measured physical activity over a one-week period. Comparisons between studies should therefore be made with caution due to the differences in study populations and measurement approaches.

This study aimed to examine the cross-sectional association between residential CO exposure and physical activity among 7-year-old children in the UK Millennium Cohort Study. Specifically, the study examined whether children living in areas with higher CO exposure differed in their objectively measured total steps and in their reported preference for outdoor sports and games, after adjustment for selected demographic and socio-economic characteristics.

## Methods

This study used a cross-sectional secondary analysis of Sweep 4 data from the MCS, linked to residential area-level MEDIx air pollution data [13].

### Study sample

The MCS is an ongoing study that includes a sample of all births in the UK for one year, beginning on 1 September 2000. So far, there have been 7 rounds of data collection involving around 19,519 children who participated in at least one of these rounds [14]. The sampling design intentionally over-represented disadvantaged areas and ethnic minority populations in England, while ensuring representation across the four UK countries [15]. The present study used data drawn from Sweep 4, when the cohort children were approximately 7 years old. Multiple data sources were merged were merged, including longitudinal family data, interview and derived variables, accelerometer-based physical activity data, and linked air pollution data obtained via the UK Data Service.

**Fig. 1:**
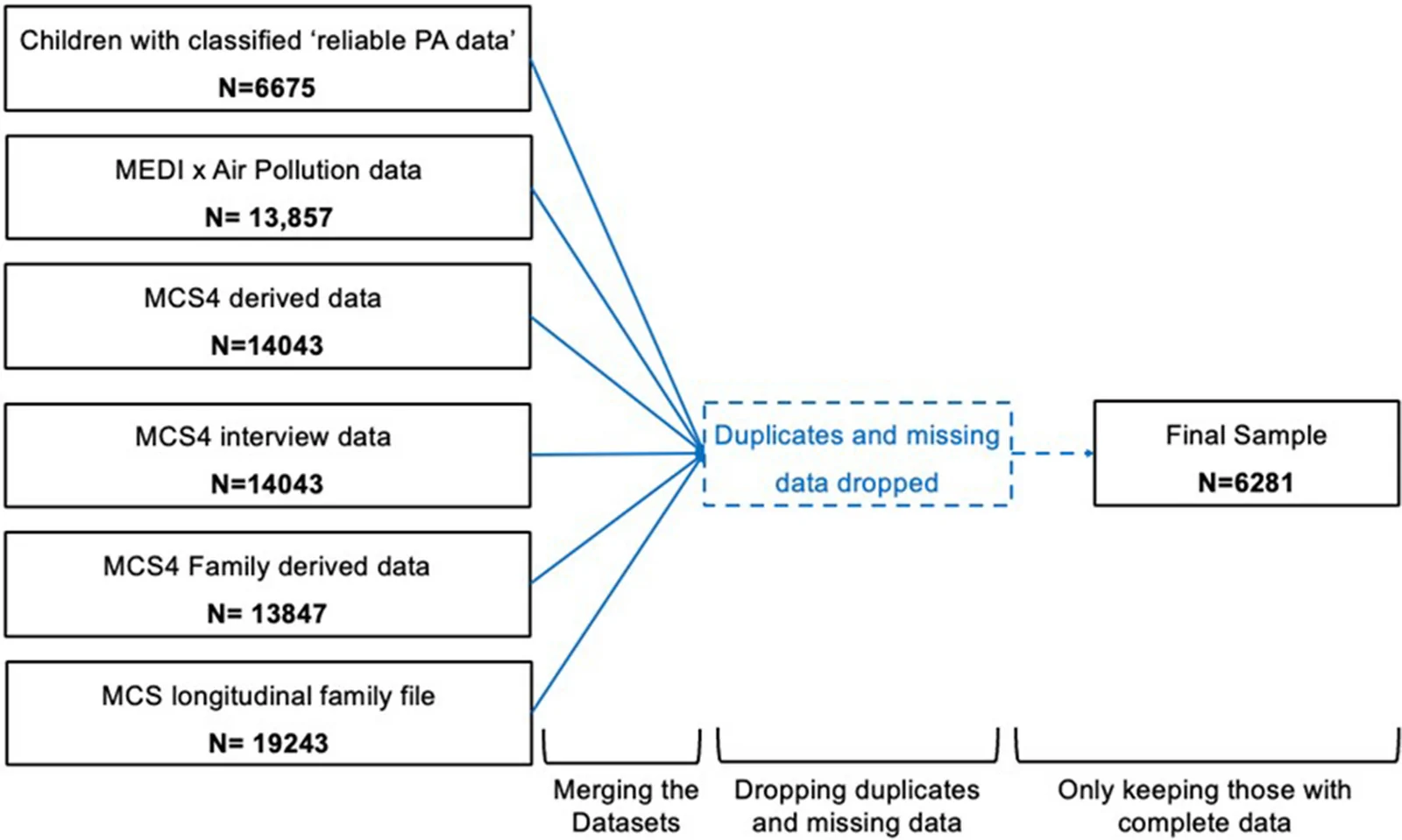
Overview of the process reaching the final sample size

The analytic sample comprised children with complete data on all variables included in the analysis, including accelerometer-derived physical activity, questionnaire responses, CO exposure, and covariates (*n* = 6,281), as shown in Fig. 1. A complete-case approach was used to ensure consistency across models. However, this approach may have introduced selection bias if children with complete data differed systematically from those excluded. Survey design and non-response weights were applied to account for the MCS sampling framework and differential participation in accelerometer data collection [16, 17], although some residual selection bias may remain.

### Exposure variables

Air pollution exposure was derived by linking residential postcodes to MEDIx data. Available pollutants included particulate matter (PM10), nitrogen dioxide (NO₂), ozone (O₃), sulphur dioxide (SO₂), and carbon monoxide (CO). These were measured as annual mean concentrations at the 2001 Census ward level using 1 km grid data derived from the National Atmospheric Emissions Inventory [18, 19]. Exposure values were converted into national deciles ranging from 1 (“least polluted”) to 10 (“most polluted”) across UK wards. Most pollutants covered the period 1999–2003, whereas CO exposure covered 2001-2006 [13, 20] 

Although multiple pollutants were available, this analysis focused on CO because its exposure period most closely aligned with the Sweep 4 physical activity data collection period. CO may also serve as an indicator of combustion- and traffic-related environmental exposures relevant to children’s everyday activity environments. In addition, area-level air pollution patterns, particularly those associated with structural factors such as traffic density and urban form, tend to remain relatively stable over time in the absence of major environmental or policy changes [18, 19]. Therefore, the CO measure is likely to capture persistent spatial variation in environmental exposure, even though it does not correspond exactly to the timing of physical activity measurement. CO exposure deciles were dichotomised into “less polluted” and “more polluted” categories to facilitate interpretation of relative differences in exposure within the dataset. However, this approach may reduce variability and obscure potential dose-response relationships, which is acknowledged in the Discussion.

### Outcome variables

**Fig. 2:**
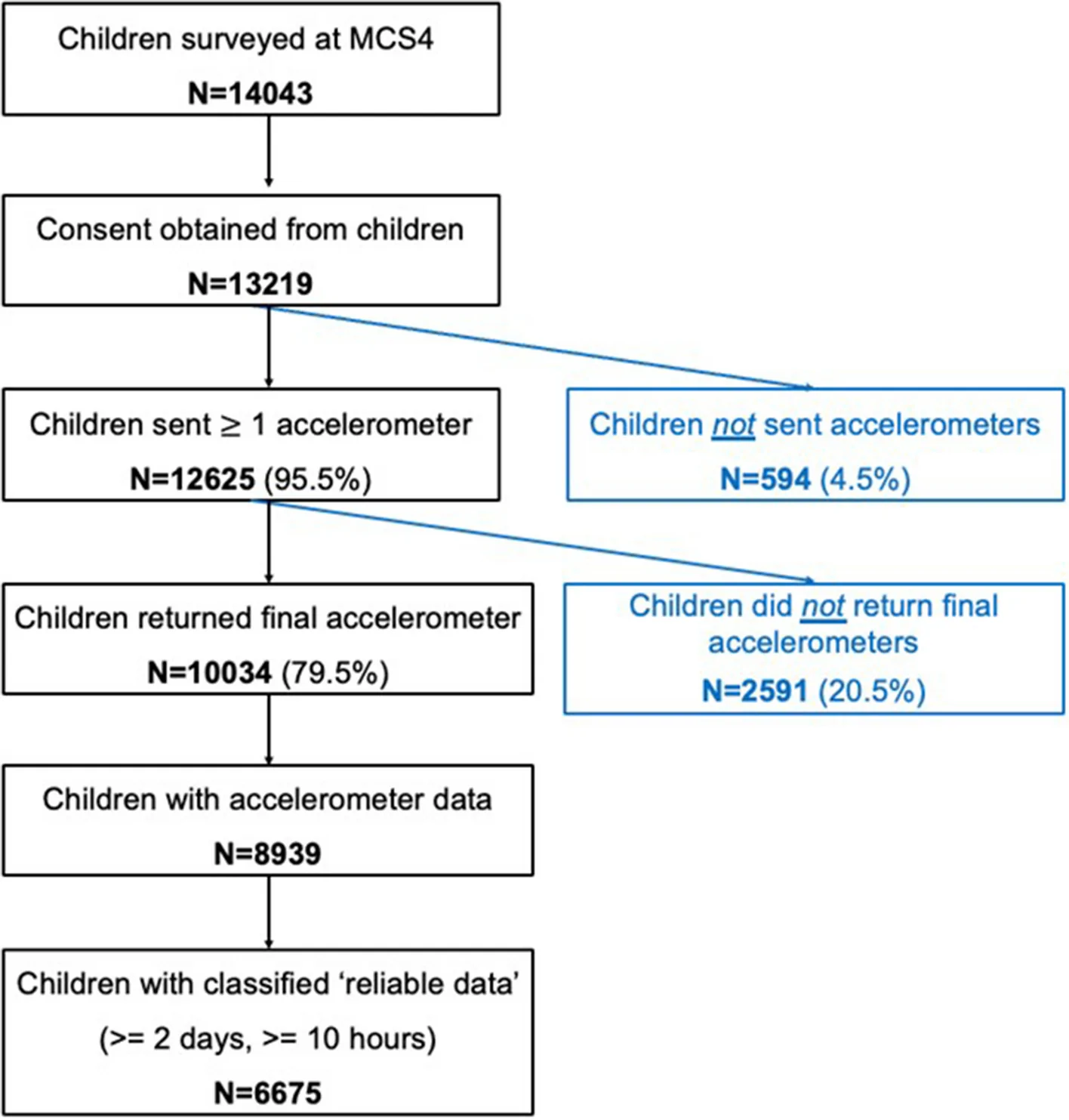
Overview of the MCS4 accelerometer data (objective data)

Children were asked to participate in the physical activity monitoring study as part of the age 7 MCS survey. This was objectively assessed using Actigraph GT1M accelerometers (May 2008-August 2009). Accelerometery is widely used for objective assessment of physical activity in children, and comprehensive information on child accelerometer procedures and wear-time criteria has been previously documented [3, 21, 22]. The MCS children wore accelerometers around their waists during waking hours for seven consecutive days, removing them only during water-based activities. A wear time of 10 h per day for at least two consecutive days was considered to provide reliable data. This established minimum wear time aligns with other large accelerometer studies in children [3]. This led to a total sample size of 6675 children with 36,309 days of observation (Fig. 2). It is worth noting that small discrepancies were identified in the demographic characteristics of the sample of children with reliable data relative to the entire cohort sample interviewed at age 7. Particularly, the boys in the South Asian and Black/Black Caribbean groups demonstrated a lower probability of having valid accelerometer data. Details regarding factors influencing reliable data collection in this study were explored by Rich et al. study [3].

The study also examined children’s self-reported preference for outdoor sports and games. Children were asked, “How much do you like to play sports and games outside?” Response options were “I don’t like it”, “I like it a little”, and “I like it a lot” [23]. For the logistic regression model, responses were dichotomised into “I like it” and “I don’t like it” to support interpretation. This measure reflects reported preference rather than objectively measured outdoor activity.

### Control variables

The analysis incorporated a limited set of demographic and socio-economic control variables available for the analytic models. Demographic and social characteristics were collected through home-based interviews at age 7 and were captured at child and family level [23]. Child-level characteristics included sex and ethnicity, categorised as White, Mixed, South Asian, Black, and other ethnic groups. Family-level socio-economic position was represented using household income/socio-economic categories derived from the MCS Sweep 4 data. This measure captures household-level socio-economic circumstances rather than area-level deprivation. Area-level deprivation was not included as an additional covariate in the final models; this is acknowledged as a limitation because neighbourhood deprivation may be associated with both residential air pollution exposure and children’s opportunities for physical activity [11].

Age was not included as a covariate because the analysis was restricted to children participating in Sweep 4 of the MCS at approximately age 7. As the analytic sample represented a narrow age band, age variation was expected to be limited. However, age would be an important covariate in studies including children across wider developmental stages.

The available models did not include several potentially relevant contextual and behavioural variables, including urban/rural location, season or weather at the time of accelerometer wear, parental education, parental physical activity, neighbourhood walkability, car ownership, school distance, housing density, or traffic density. These omissions are acknowledged as potential sources of residual confounding in the Discussion and Limitations sections. A justification can be found in the Appendix Section (Appendix 1). Key summary statistics are presented in Table 1.

**Table 1:** Descriptive Statistics Table, including a breakdown of the variables used in this study

**Variable**	**N**	**Unweighted data Mean/(s.d)** % of total	**Weighted data** **Mean/(s.d)** % of total
All Children	**6,281**		
Child’s gender			
Male	3,067	48.8%	50.8%
Female	3,214	51.2%	49.2%
Child’s ethnicity			
White	5,538	88.2%	85.6%
Mixed	156	2.5%	3.1%
South Asian	366	5.8%	6.8%
Black	147	2.3%	3.1%
Other	74	1.2%	1.4%
Child’s SEP			
Lowest quintile	899	14.3%	20.1%
Second quintile	1,105	17.6%	19.8%
Third quintile	1,341	21.3%	19.9%
Fourth quintile	1,487	23.7%	20.5%
Highest quintile	1,450	23.1%	19.7%
Carbon Monoxide			
Less polluted half	2,326	37.0%	33.8%
Most polluted half	3,955	63.0%	66.2%
Total Steps		10,406.71 (3558.28)	1053.82 (3622.83)
How much do you like playing sports and games outside?			
I like it	4,481	71.3%	71.5%
I don’t like it	1,800	28.7%	29.5%

### Statistical analysis

Two models were used in this study. The first model examined the association between CO exposure and total steps. The second model examined the association between CO exposure and preference for outdoor sports and games. Both models used survey design and non-response weights from the MCS4 and physical activity monitoring data to reduce potential sampling and non-response bias [16, 17]. For the first model, the study employed regression modelling to predict the relationship between the explanatory variables and changes in the outcome (total steps). This model was chosen as it can assess the impact of the categorical exposure (CO) on the continuous outcome while controlling for covariates. Regression models enable estimation of coefficients that illustrate the relationship between the exposure and the outcome, providing a quantitative assessment. The second model used logistic regression to analyse explanatory variables and their influence on changes in the outcome (“how much do you like playing sports and games outdoors?”). This modelling approach provides odds ratios that measure the strength and direction of associations between these categorical variables. Logistic regression also accounts for multiple confounding variables, helping control for potential sources of bias and assess the individual influence of each categorical factor on the outcome variable. The capability to analyse multiple variables makes logistic regression a crucial tool for examining complex relationships between categorical predictors and outcomes. All tests were tested at a 5% confidence level. Analyses were conducted using STATA/SE V.18 for statistical computing.

## Results

The study had a total of 6,281 participants. Table 1 presents the analytic sample characteristics, including sample sizes and percentages for the exposure, outcome, and covariate variables included in the complete-case analysis. Only those with all the characteristics required in this study were included in the complete case analysis. The study participants come from a range of socio-economic backgrounds, with 14.3% living in the lowest quintile, 17.6% in the second, 21.3% in the third, 23.7% in the fourth, and 23.1% in the highest. Most participants were ethnically white (88.2%) compared with those from other ethnic backgrounds (11.8%). Further, the sample was quite evenly distributed for participants’ gender with 48.8% male and 51.2% female.

In the first model (Table 2), those living in areas exposed to higher CO levels take an average of 285.68 more steps per day than those living in the less-polluted half. The effect is statistically significant (*p*-value = 0.027), the coefficient is positive, and the standard error of this estimate is 128.64, indicating a consistent positive relationship between pollution level and steps-based physical activity. Once adjusted for the covariates (Table 3), individuals living in higher-exposed areas exhibited 356.26 more steps in their total step count compared to those in the lower category, with a significant *p*-value of 0.007. The R-squared value (0.052) indicates that the model explains approximately 5.21% of the variance in total steps. Furthermore, F = 16.62 and *p* < 0.001 indicate that the model is statistically significant and that the predictors are related to the dependent variable. Females are associated with a lower total of steps than males, with *p* < 0.001, indicating a strong, significant negative association. Similarly, compared with the baseline group (individuals living in the lowest socio-economic quintile), all other income categories are associated with lower total steps, with *p*-values indicating strong statistical significance. Interestingly, South Asians are the only group with a significantly lower level of about 1197.87 units in steps (*p*-value < 0.001). Being female was associated with fewer total steps than being male. Compared with the reference socio-economic group, children in higher-income categories also recorded lower total step counts. South Asian children recorded significantly fewer steps than children in the White reference group.

**Table 2:** Model 1, regression model examining the association between carbon monoxide on total steps

Total Steps	Coef	Std.err	t-value	*p*-value	[95% conf	interval]	sig
Carbone Monoxide	356.26	131.40	2.71	0.007	97.90	614.62	**
Constant	11,722.36	228.23	51.36	< 0.001	11,273.64	12,171.08	***
Mean dependent var		10,503.82	SD dependent var			3622.83	
R-squared		0.001	Number of obs			6,281	
F-test		4.93	Prob > F			0.027	
Akaike crit. (AIC)		120,270.3	Bayesian crit. (BIC)			120,344.4	

^***^p < 0.001

^**^p < 0.01

^*^p < 0.05

**Table 3:** Model 1, regression model examining the association between carbon monoxide on total steps, adjusted for covariates (child’s gender, ethnicity, and socio-economic position)

Total Steps	Coef	Std.err	t-value	*p*-value	[95% conf	interval]	sig
Carbone Monoxide	356.26	131.40	2.71	0.007	97.90	614.62	**
Child’s gender							
Female	−1423.97	116.24	−12.25	< 0.001	−1652.51	−1195.43	***
Child’s ethnicity							
Mixed	−702.71	369.45	−1.90	0.058	−1429.08	23.66	
South Asian	−1197.87	283.14	−4.23	< 0.001	−1754.56	−641.18	***
Black	−282.59	722.12	−0.39	0.696	−1702.33	1137.15	
Other	−452.58	338.91	−1.34	0.183	−1118.90	213.74	
Child’s SEP							
Second quintile	−722.31	222.96	−3.24	0.001	−1160.66	−283.95	**
Third quintile	−924.47	208.98	−4.42	< 0.001	−1335.34	−513.59	***
Fourth quintile	−733.49	227.14	−3.23	0.001	−1180.06	−286.91	**
Highest quintile	−805.25	224.89	−3.58	< 0.001	−1247.41	−363.09	***
Constant	11,722.36	228.23	51.36	< 0.001	11,273.64	12,171.08	***
Mean dependent var		10,503.82	SD dependent var			3622.83	
R-squared		0.052	Number of obs			6,281	
F-test		16.62	Prob > F			< 0.001	
Akaike crit. (AIC)		120,270.3	Bayesian crit. (BIC)			120,344.4	

^***^p < 0.001

^**^p < 0.01

^*^p < 0.05

In the second model (Table 4), those living in more polluted locations are, on average, 20% more likely than those living in less polluted locations to report enjoying being outside (OR = 1.20; *p* = 0.012). Once adjusted for the covariates (Table 5), individuals living in more polluted areas are 1.19 times (19%) more likely to play sports and games outside than those living in less polluted areas (*p* = 0.035). This suggests a slightly higher likelihood of liking outdoor activities with higher carbon monoxide exposure, although the confidence interval (1.01 to 1.39) indicates that the effect size and significance are modest. Nevertheless, the F-statistic and associated *p*-values (Tables 4 and 5) suggest that the overall models are statistically significant. Females were 1.53 times more likely to answer that they like playing sports and games outside than males, which is highly significant at *p* < 0.001, indicating stronger preferences for outdoor activities among females in this sample. Individuals living in the second to fifth quintile show odds ratios below 1, indicating a lower likelihood of liking outdoor activities than the lowest income bracket (first quintile), though only the fourth quintile’s results are significant (*p* = 0.046). Finally, none of the ethnicity categories has odds ratios statistically significantly different from the reference category (those from white ethnic groups). This association was modest and should be interpreted cautiously, particularly because the confidence interval was close to the null value. Being female was associated with higher odds of reporting a preference for outdoor sports and games compared with being male.

**Table 4:** Model 2, logistic regression model examining the association between carbon monoxide on children’s preferences for doing outdoor sports and games

How much do you like playing sports and games outside?	Odds ratio	Std.err	t-value	*p*-value	[95% conf	interval]	sig
Carbone Monoxide	1.20	.089	2.52	0.012	1.42	1.393	*
Constant	.337	.022	−16.88	< 0.001	.329	.415	***
F-test		6.36	Prob > F			0.0121	

^***^p < 0.001

^**^p < 0.01

^*^p < 0.05

**Table 5:** Model 2, logistic regression model examining the association between carbon monoxide on children’s preferences for doing outdoor sports and games, adjusted for covariates

How much do you like playing sports and games outside?	Odds ratio	Std.err	t-value	*p*-value	[95% conf	interval]	sig
Carbone Monoxide	1.19	.095	2.12	0.035	1.01	1.39	*
Child’s gender							
Female	1.53	.120	5.47	< 0.001	1.31	1.79	***
Child’s ethnicity							
Mixed	.947	.195	−0.26	0.793	.632	1.420	
South Asian	.954	.167	−0.27	0.786	.676	1.35	
Black	1.30	.337	1.01	0.312	.780	2.16	
Other	.645	.235	−1.20	0.229	.315	1.32	
Child’s SEP							
Second quintile	.911	.111	−0.76	0.445	.716	1.16	
Third quintile	1.00	.133	−0.03	0.975	.774	1.30	
Fourth quintile	.790	.093	−2.00	0.046	.626	.996	*
Highest quintile	.839	.109	−1.35	0.179	.650	1.08	
Constant	.332	.043	−8.49	< 0.001	.257	.4284	***
F-test		4.82	Prob > F			< 0.001	

^***^p < 0.001

^**^p < 0.01

^*^p < 0.05

## Discussion

This study examined whether average residential CO exposure was associated with objectively measured physical activity and reported preference for outdoor sports and games among 7-year-old children in the UK. Higher CO exposure was modestly associated with higher total steps and slightly higher odds of reporting a preference for outdoor activities. However, these findings should be interpreted with caution.

The exposure and outcome measures were not contemporaneous, although, as described in the Methods, the CO measure is likely to reflect longer-term spatial differences in air pollution exposure. Further, the explanatory power of the adjusted model is central to interpretation. The model explained approximately 5.2% of the variance in total steps, indicating that most variation in children’s physical activity was not captured by the variables included. Physical activity in children is shaped by a wide range of influences, including family routines, school travel, seasonality, weather, neighbourhood design, access to outdoor space, and parental activity. Previous research has also highlighted the importance of parental influence in shaping children’s physical activity behaviours [24, 25]. Taken together, the observed association between CO exposure and total steps is modest and should not be interpreted as evidence of a meaningful determinant of children’s physical activity. Comparison with previous studies requires caution because the exposure and outcome measures differ substantially. Smith et al. [26] examined neighbourhood environmental characteristics, including NO₂, in relation to accelerometer-measured physical activity among adults, whereas Wells et al. [12] examined self-reported activity change in response to perceived or reported poor air quality [12, 26]. By contrast, the present study examined residential CO exposure in relation to children’s one-week accelerometer-measured total steps and self-reported outdoor play preference. These methodological differences mean that the present findings should not be interpreted as directly contradicting previous studies. Rather, they suggest that associations may vary by pollutant, age group, exposure measurement, behavioural outcome, and contextual factors. The CO exposure variable used in this analysis was based on relative deciles across UK wards rather than absolute pollutant concentrations [13]. Therefore, the “more polluted” category should be interpreted as relatively higher CO exposure within the linked dataset, not necessarily as exposure above a health-based national or international threshold. This limits direct comparison with WHO guideline values or other international measures of harmful air pollution [27].

The unexpected positive association between higher CO exposure and total steps is unlikely to reflect a direct behavioural effect of air pollution. A more plausible explanation is that CO exposure is capturing broader neighbourhood characteristics. Areas with higher CO exposure are often more urban or traffic-dense and may also offer shorter distances to school, greater walkability, more local destinations, and different opportunities for outdoor play [28]. These contextual factors may increase children’s daily steps independently of pollution exposure. In the absence of direct measures of urbanicity, walkability, traffic density, school distance, housing density, parental activity, or car ownership, residual confounding is likely.

In addition, children and their families are unlikely to base activity decisions on long-term air pollution exposure, particularly when it is not visible or explicitly communicated. Annual residential air pollution measures do not necessarily correspond to visible pollution or day-to-day perceptions of environmental risk. While previous studies have examined behavioural responses to perceived poor air quality [12], the present study did not measure perception, awareness, or behavioural adaptation. Therefore, the findings should not be interpreted as evidence that children or parents consciously modified physical activity behaviour in response to air pollution exposure. Children’s physical activity is also shaped by routine structures such as school travel, playground time, organised activities, and family routines, rather than deliberate responses to environmental exposure. The observed association between higher CO exposure and total steps is therefore more consistent with characteristics of urban and walkable environments than behavioural responses to pollution.

Being female was associated with lower total steps compared with being male, although girls had higher odds of reporting a preference for outdoor sports and games. This suggests that reported preference for outdoor play and objectively measured physical activity may capture different aspects of behaviour. Preferences may not translate directly into total activity, particularly where opportunities for activity are shaped by parental rules, school routines, safety perceptions, social norms, or access to outdoor spaces. Previous studies have also reported sex differences in physical activity among children [29], and parental correlates of outdoor play may differ for boys and girls [30].

Socio-economic position was also associated with children’s total steps, although interpretation is complex. Household socio-economic circumstances may shape children’s physical activity through access to organised activities, school travel patterns, neighbourhood safety, housing conditions, car ownership, parental working patterns, and access to outdoor space. At the same time, socio-economic conditions may be associated with residential environmental exposure [11, 31]. As area-level deprivation and detailed built-environment measures were not included, these findings should be interpreted cautiously.

Similarly, although ethnicity was not associated with reported preference for outdoor activity, South Asian children recorded lower total steps than children in the White reference group. This should be interpreted cautiously, given the limited representation of some groups and the likelihood that differences reflect broader social, cultural, and environmental factors not captured in the present analysis. Future research using more diverse samples and richer contextual data is needed.

### Strengths and limitations

This study has several strengths. It used a large UK child cohort and included objectively measured physical activity, reducing reliance on self-reported activity data. The use of linked environmental exposure data also enabled examination of residential CO exposure in relation to children’s physical activity and outdoor play preferences [13]. In addition, the analysis applied survey and non-response weights to account for the MCS sampling design and accelerometer non-response [16, 17].

This study has several limitations that affect interpretation. First, the cross-sectional design precludes causal inference. The findings should therefore be interpreted as associations between residential CO exposure and physical activity outcomes, not as evidence that CO exposure changes children’s activity behaviour. Second, there was a temporal mismatch between the exposure and outcome measures. CO exposure was measured as average residential area-level exposure over an extended period, whereas physical activity was recorded over one week using accelerometry. This may have introduced exposure misclassification if pollution levels during the accelerometer week differed from the longer-term average. Third, there was potential spatial mismatch because children’s physical activity may have occurred outside the residential neighbourhood, including at school, during travel, or in other neighbourhoods. Residential CO exposure may therefore not fully represent the pollution exposure experienced during the week of physical activity monitoring. Fourth, the models did not include several potentially important confounders, including urban/rural location, season, weather, parental education, parental physical activity, car ownership, school distance, neighbourhood walkability, traffic density, housing density, or area-level deprivation. These omitted factors may explain part of the observed association between CO exposure and physical activity. Fifth, the complete-case analysis may have introduced selection bias. Children with valid accelerometer data and complete covariate information may differ from those excluded in ways related to physical activity, socio-economic circumstances, ethnicity, or residential environment. Although survey and non-response weights were applied, these may not fully account for all differences between included and excluded participants [16, 17]. Sixth, dichotomising CO exposure into “less polluted” and “more polluted” halves improved interpretability but may have reduced information and obscured dose-response patterns across the original deciles. Future research could examine pollutant exposure using more granular categories or continuous exposure measures where available. Finally, awareness of wearing an accelerometer may have affected recorded physical activity levels overall. However, there is no clear reason to expect this reactivity to differ systematically by residential CO exposure category. Therefore, this issue is likely to be less important for the exposure-outcome association than the temporal and spatial exposure limitations described above.

## Conclusion

This study examined the cross-sectional association between residential carbon monoxide exposure, objectively measured physical activity, and preference for outdoor sports and games among 7-year-old children in the UK Millennium Cohort Study. Higher residential CO exposure was modestly associated with higher total steps and slightly higher odds of reporting a preference for outdoor sports and games. However, these findings should be interpreted cautiously. The adjusted model explained only a small proportion of the variation in total steps, and the observed associations may reflect residual confounding by urbanicity, neighbourhood walkability, school travel, traffic density, housing density, deprivation, family characteristics, or other contextual factors not captured in the analysis.

The findings do not suggest that CO exposure promotes physical activity. Rather, they highlight the complexity of examining children’s physical activity in relation to environmental exposures using cross-sectional data. Future studies should examine multiple pollutants, use more temporally aligned exposure and activity data, and include richer measures of the built environment, school travel, weather, parental behaviours, household characteristics, and area-level deprivation. This would help clarify whether observed associations reflect pollutant exposure itself or the broader social and environmental contexts in which children live.

## Supplementary Information

Supplementary Material 1.

## Data Availability

This study utilises secondary data from the Millennium Cohort Study (MCS), including the core MCS dataset and a derived dataset, Millennium Cohort Study, Sweeps 1–6, 2001–2015: Linked to MEDIx Air Pollution Deciles, which is available through the UK Data Service.
