# Supplementary material for: Associations between carbon monoxide exposure, physical activity, and outdoor play preferences among UK children: evidence from the millennium cohort study
Source: BMC Public Health. 2026 Jun 27;26:2824. doi: 10.1186/s12889-026-28149-8 (PMC13644019; doi:10.1186/s12889-026-28149-8)
Supplement: Supplementary file 1 [file 12889_2026_28149_MOESM1_ESM.docx]

| **Appendix 1:** Bivariate associations between exposure, outcome, and covariates | | | | | | | | | | |
| --- | --- | --- | --- | --- | --- | --- | --- | --- | --- | --- |
| **Variable** |  | **Unweighted data** | | **Weighted data** | **Carbon Monoxide (cat.)** | | **Total Steps (cont.)** | | **How much do you like playing sports and games outside?  (cat.)** | |
|  | **N** | **Mean/(s.d)**  % of total | **Mean/(s.d)**  % of total | | **chi2** | **P-value for chi2** | **Mean/(s.d)** | **P-value for t-test** | **chi2** | **P-value for chi2** |
|  |  |  |  | |  |  |  |  |  |  |
| **Child’s gender** |  |  |  | | **0.002** | **0.967** | . | **0.001** | 45.58 | **0.001** |
| Male | 3,067 | 48.83% | 50% | | . | . | 11117.29 (3827.06) | . | . | . |
| Female | 3,215 | 51.17% | 49% | | . | . | 9728.62 (3135.15) | . | . |  |
|  |  |  |  | |  |  |  |  |  |  |
|  |  |  |  | | **chi2** | **P-value for chi2** | **Mean/(s.d)** | **P-value for Anova** | **chi2** | **P-value for chi2** |
| **Child’s ethnicity** |  |  |  | | **399.03** | **0.001** | **.** | **0.016** | **11.38** | **0.023** |
| White | 5,538 | 88.17% | 85.63% | | . |  | 10474.67  (3569.75) | . | . | . |
| Mixed | 156 | 2.48% | 3.1% | | . |  | 9941.07 (3414.17) | . | . | . |
| South Asian | 366 | 5.83% | 6.81% | | . |  | 9789.33 (3175.38) | . | . | . |
| Black | 147 | 2.34% | 3.08% | | . |  | 10152.88 (3871.56) | . | . | . |
| Other | 74 | 1.18% | 1.39% | | . |  | 9859.80  (3790.09) | . | . | . |
|  |  |  |  | |  |  |  |  |  |  |
|  |  |  |  | | **chi2** | **P-value for chi2** | **Mean/(s.d)** | **P-value for t-test** | **chi2** | **P-value for chi2** |
| **Child’s SEP** |  |  |  | | **77.99** | **0.001** | . | **0.001** | **5.9401** | **0.20** |
| Lowest quintile | 899 | 14.31% | 20.12% | | . | . | 10770.04  (3852.08) | . | . | . |
| Second quintile | 1,105 | 17.59% | 19.76% | | . | . | 10433.35  (3691.44) | . | . | . |
| Third quintile | 1,341 | 21.35% | 19.93% | | . | . | 10266.48 (3602.93) | . | . | . |
| Fourth quintile | 1,487 | 23.67% | 20.49% | | . | . | 10356.39 (3417.06) | . | . | . |
| Highest quintile | 1,450 | 23.08% | 19.7% | | . | . | 10342.38 (3349.31) | . | . | . |
|  |  |  |  | |  |  |  |  |  |  |

As demonstrated in **Appendix 1**, ethnicity has a significant association with carbon monoxide (p=0.001), total steps (p=0.016), and the preferences of playing sports and games outside (p=0.023). This suggests that ethnicity can be included in the final analytical sample as it demonstrates statistical significance. Next, the child’s socio-economic position (SEP), there is a significant association with carbon monoxide levels (p=0.001) and total steps (p=0.001), suggesting SEP could be a confounder for these outcomes but not for the preferences of playing sports and games outside (p-value for chi2 = 0.20). However, since SEP is considered a confounder in its relationship with the total steps taken, this study will continue to adjust for SEP in our statistical models. This approach ensures consistency and comparability between the two analyses as our study focuses on examining two outcomes: total steps and preferences of playing sports and games outside. The decision to adjust for SEP, rather than stratify by it, is made to maintain a uniform methodological framework across our research inquiries. Furthermore, child’s gender demonstrates an association with total steps (p=0.001) and the preferences of playing sports and games outside (p=0.001), but not with carbon monoxide (p=0.967). Therefore, the impact of child’s gender on carbon monoxide levels was assessed through an interaction term to see whether gender can be considered an effect modifier. The interaction between CO and gender while looking at the relationship between outcome (total steps) and exposure (CO) was not significant (p-value > 0.05). This was also the case once we took the preferences of playing sports and games outside as our outcome (p-value > 0.05). This suggests that child’s gender cannot be considered as an effect modifier. Nonetheless, this study included gender in the final analytical model as it may be a confounder for other association examined in the model (total steps and preferences on playing sports and games outside). Indeed, if gender is associated with other variables that are related to the outcome, it can potentially impact these relationships if not accounted for. Additionally, it is common for ethical and reporting standards in research to include important factors such as gender. This ensures that data analysis properly considers the fundamental demographic characteristics.
